# Risk behaviors for infectious Keratitis among contact lens wearers in Switzerland: an analysis by contact lens type

**DOI:** 10.1016/j.optom.2026.100622

**Published:** 2026-06-27

**Authors:** Philipp Perschak, Sadiq Said, Simone Metzler, Sandrine Anne Zweifel, Daniel Barthelmes, Dominique Hermann, Didier Herrmann, Farhad Hafezi, Léonard Kollros, Martina M Bosch, Frank Blaser

**Affiliations:** a Department of Ophthalmology, University Hospital Zurich, University of Zurich, Zurich, Switzerland; b Eye Clinic Wettingen, Wettingen, Switzerland; c Department of Ophthalmology, Cantonal Hospital St. Gallen, St. Gallen, Switzerland; d Institute of Optometry, FHNW University of Applied Sciences and Arts Northwestern Switzerland, Olten, Switzerland; e ELZA Institute, Zurich, Switzerland

**Keywords:** Contact lens, Survey, Infectious keratitis, Risk factors, Risk behavior

## Abstract

**Background:**

To evaluate risk behaviors for infectious keratitis among contact lens (CL) wearers in Switzerland, focusing on survey-based, observational subgroup analyses by contact lens type.

**Methods:**

This investigator-initiated, cross-sectional, survey-based, observational, multi-center study was conducted across five eye care institutions in Switzerland. Between August 2023 and August 2024, we interviewed participants wearing CLs using a structured questionnaire. We assigned four subgroups, which included daily disposable soft contact lenses (SCL), reusable SCL, rigid corneal contact lenses (RCL), and rigid scleral contact lenses (ScCL). The survey collected data on demographics, CL type and indication, self-assessed CL knowledge, and risk behaviors related to infectious keratitis. We classified risk behaviors into three main categories, including hand hygiene, wearing behavior, and exposure to water or saliva.

**Results:**

156 participants were included in the final analysis. Regarding the subgroup allocation, 38 of 156 (24.4%) wore daily disposable SCLs, 48 (30.8%) reusable SCLs, 40 (25.6%) RCLs, and 30 (19.2%) ScCLs. Overall, participants wearing rigid CLs felt better informed about CL care and demonstrated fewer risk behaviors than SCL users. However, risk behaviors were observed across all subgroups, including applying or removing lenses with potentially contaminated hands, extended CL wear, sleeping with CLs, and exposure to water or saliva.

**Conclusion:**

This study revealed variations in hygiene practices, wearing behavior, exposure to water or saliva, and risk perception across different CL types. Nevertheless, risk factors for infectious keratitis were present among all subgroups, underscoring the need for lens-specific patient education under professional supervision to improve compliance and mitigate complications.

## Introduction

Since their introduction in the 1970s, soft contact lenses (SCL) have revolutionized refractive error correction, providing a convenient alternative to spectacles.^1^^,^^2^ Further technological advances led to a diverse range of contact lens (CL) types, tailored to accommodate the customers’ specific needs and lifestyle preferences.^3^, ^4^, ^5^, ^6^ These include daily disposable SCLs, reusable SCLs with various replacement schedules, and rigid CLs such as rigid corneal contact lenses (RCL) and rigid scleral contact lenses (ScCL).

Globally and with an increasing trend, over 140 million people rely on CLs, whereby daily disposable SCLs are most commonly used.^6^, ^7^, ^8^, ^9^ This global trend is also observed in Switzerland. According to a 2021 survey by the Swiss Association of Opticians, 24.9% of individuals aged 16 to 74 reported wearing CLs either exclusively or in combination with glasses.^10^ Furthermore, there has been a marked increase in both overall CL wear and the preference for daily disposable SCLs, while rigid CLs account for a small market fraction.^11^ However, despite their benefits and development, CLs still present foreign bodies on the ocular surface and pose an inherent risk to ocular health, especially when proper lens care is not provided. Vision-threatening complications such as infectious keratitis, which may result in corneal scarring, may ensue. Thus, it comes as no surprise that CL-wear remains the leading predisposing factor for infectious keratitis in developed countries.^12^ Overall, annualized incidence rates per 10′000 years (95% Confidence Interval) have been reported as 2.2 to 4.1 for daily and 9.3 to 20.9 for extended CL wear.^13^ Corneal opacities, including those from infectious keratitis, are still ranked, according to the World Health Organization, as the fifth leading cause of global blindness.^14^, ^15^, ^16^

The CL type, the user’s age and gender, and lifestyle factors such as smoking are considered risk factors for CL-related infectious keratitis.^17^ However, overall CL care is the most critical risk factor.^18^ Based on the literature, the risk factors for infectious keratitis related to CL care can be divided into three main categories. First, insufficient hand hygiene related to CL wear has been shown to increase the risk of keratitis.^19^ Wearing behavior as a second category can further be divided into risk factors such as excessive or overnight wear.^18^^,^^20^ Contact to potentially contaminated water (e.g. tap water, and other sources such as lake or seawater), as well as saliva can be discussed as a third risk category.^21^, ^22^, ^23^ Despite emphasis on patient education, only 0.3% of daily and 2.7% of extended CL wearers are fully compliant regarding CL care.^24^ Additionally, compliance rates vary in the literature when looking at specific risk factors, such as hand hygiene, with reported incompliance rates between 11.2% and 52.4%, or water exposure that has been reported with rates of 25.6% up to 72% in some studies.^25^, ^26^, ^27^, ^28^

While studies emphasize the importance of CL care and education, a notable research gap concerning CL-related risk behavior exists in Switzerland. This survey-based, observational study aims to investigate risk behaviors associated with the aforementioned risk categories among different CL types in Switzerland. Exploring this matter may help develop targeted interventions and educational strategies to enhance patient compliance and reduce the incidence of CL-related complications in Switzerland.

## Methods

This investigator-initiated, cross-sectional, survey-based, observational, multi-center study was conducted across multiple eye care institutions in Switzerland, including the University Hospital Zurich, Cantonal Hospital St. Gallen, the University of Applied Sciences and Arts Northwestern Switzerland, the ELZA Institute, and the Eye Clinic Wettingen. Using an observational survey, we explored the risk behavior of CL wearers between August 2023 and August 2024. The leading ethics committee reviewed the study protocol and declared no objection (BASEC number Req-2023–00437). Nevertheless, we obtained verbal informed consent from all participants for the use of their data, which were handled according to Good Clinical Practice.

We selected participants at random during routine or emergency appointments at designated recruitment centers. We included individuals of all ages who applied any CLs for any indication and interviewed underage participants in the presence of their legal guardian. Surveys were conducted in an undisturbed environment, either in person or by phone. Each participating center had designated interviewers (PP, SM, DM, DH, LK, and MKB), all of whom received uniform instructions in the form of a private meeting discussing the survey in total prior to conducting the interviews to ensure a standardized approach to interviews and comparability. Choosing an appropriate sample size in any qualitative research is a debatable topic.^29^ Having in mind a broad set of indications and robust dataset, we aimed to include 150 participants. Initially, we collected patient characteristics, including age, gender, CL type, CL indication, and years of use. Subsequently, we asked open-ended, single- or multiple-choice questions addressing their self-assessed CL knowledge and the three aforementioned risk categories related to overall CL care, including hand hygiene, wearing behavior, and exposure to potentially contaminated water and saliva. Investigators did not provide any further guidance, and we did not record the surveys. To ensure consistency, we categorized the responses based on predefined answer options, which we ticked as soon as participants mentioned them. Additionally, we took field notes in cases of unclear or novel responses. To anonymously input the data, we applied an iSurvey-based questionnaire (Harvest Your Data, Wellington, New Zealand). Participants were allocated into four prespecified categories based on CL type: daily disposable SCLs, reusable SCLs, RCLs, and ScCLs. Participants who were mixing different CL types or applying other CLs at the time of the interview, such as cosmetic lenses, were excluded from the subgroup analysis.

We used descriptive statistics, presenting continuous data as means with standard deviation and medians with interquartile ranges (IQR) or minimum to maximum values. Categorical data were reported as frequencies and percentages. No formal weighting system for the individual risk factors was applied. All statistical analyses were performed using Microsoft Excel (Microsoft Corporation, Redmond, Washington, USA), and figures were created using Prism version 10.2.3 (GraphPad Software, San Francisco, California, USA).

## Results

### Participant demographics

**Fig. 1:**
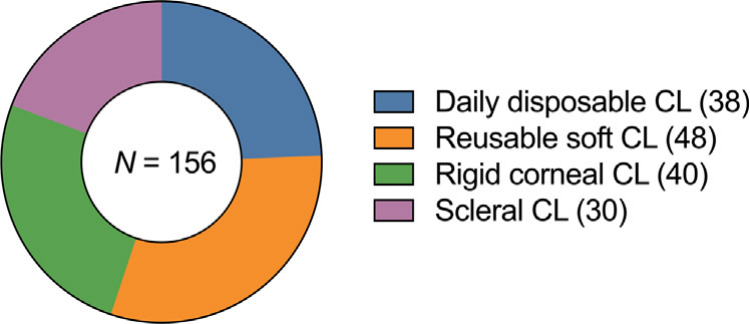
Donut parts of whole chart illustrating the distribution of contact lens types among study participants. CL = contact lens.

Between August 2023 and August 2024, we interviewed a total of 172 CL wearers, of whom 156 (90.7%) were allocated into one of the four subgroups. The median (IQR [range]) age was 39.5 (27 - 54 [10 - 82]) years, and 74 (47.4%) participants were female. Among the 156 participants, 38 (24.4%) wore daily disposable SCLs, 48 (30.8%) reusable SCLs, 40 (25.6%) RCLs, and 30 (19.2%) ScCLs (Fig. 1). None of the participants suffered from CL-associated infectious keratitis. Although orthokeratology wearers were initially screened, only a single eligible participant was identified and excluded from the final analysis due to insufficient sample size. The median (IQR [range]) duration of CL use was 15 (6 - 28 [0.1 - 70]) years. Regarding the CL indication, a multiple-choice format was applied, in which 93 (59.6%) participants reported wearing CLs for myopia, 8 (5.1%) for hyperopia, and 28 (17.9%) for astigmatism. Additionally, 48 (30.8%) participants wore CLs due to keratoconus, and 18 (11.5%) cited other reasons, including corneal ectasia other than keratoconus, severe surface wetting disorder, aphakia or trauma. Table 1 provides a comprehensive overview of participant demographics.

**Table 1:** Participant characteristics. Data are presented as numbers (%), mean ± standard deviation, or median (interquartile range [range]). SD = standard deviation; IQR = interquartile range.

Patients in subgroup analysis, n	156
Sex, female	74 (47.4%)
Age in years	
Mean ± SD	41.6 ± 16.2
Median (IQR [range])	39.5 (27 - 54 [10 - 82])
Duration of using contact lenses, years	
Mean ± SD	18.4 ± 15.0
Median (IQR [range])	15 (6 - 28 [0.1 - 70])
Indication	
Myopia	93 (59.6%)
Hyperopia	8 (5.1%)
Astigmatism	28 (17.9%)
Keratoconus	48 (30.8%)
Other (e.g. other corneal ectasia, surface wetting disorder, aphakia)	18 (11.5%)

### Self-assessment of knowledge

**Fig. 2:**
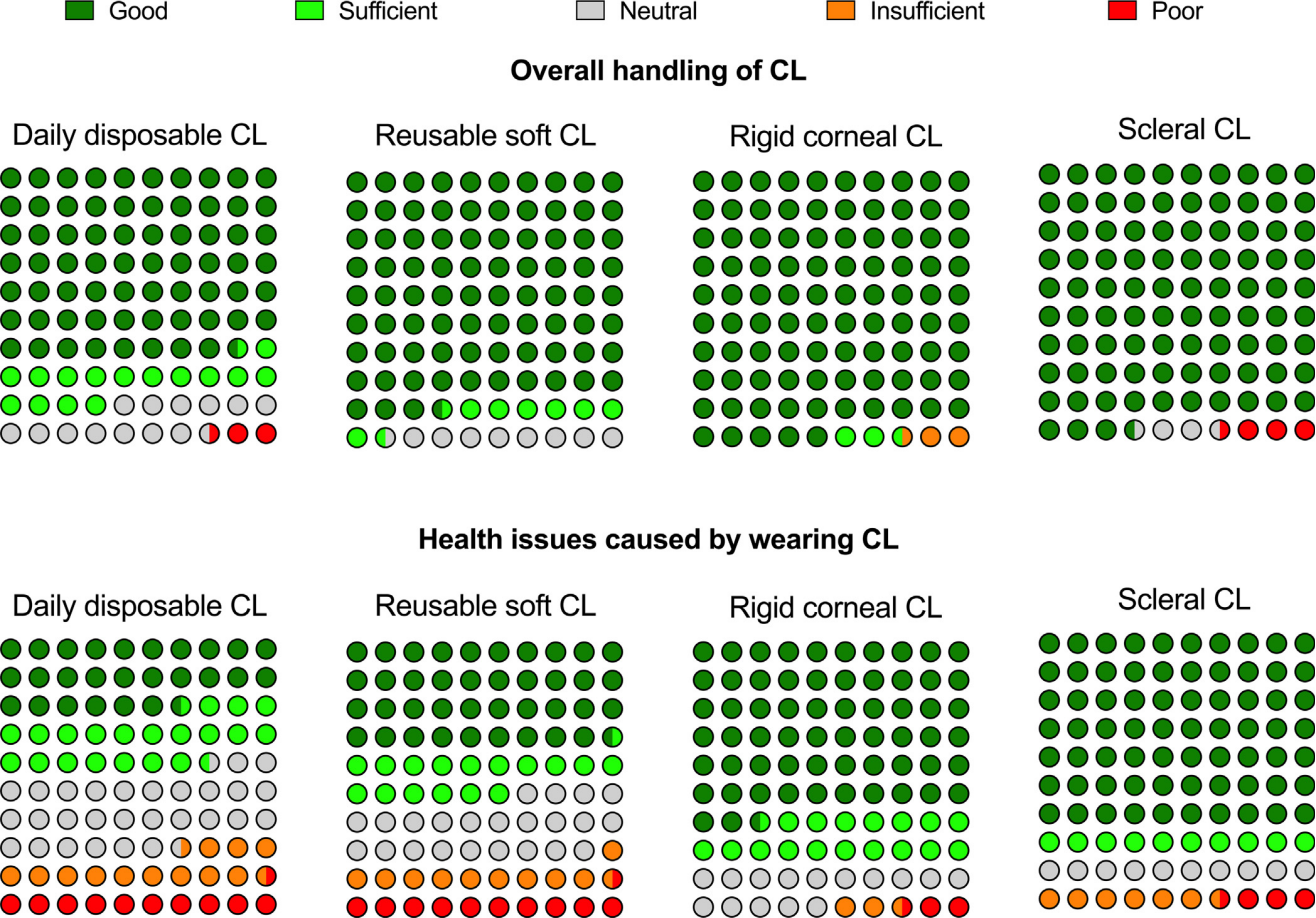
Parts of whole 10×10 dot plots depicting the single-choice responses by contact lens subgroup regarding general contact lens handling (top) and awareness of lens-related health issues (bottom). CL = contact lens.

Participants rated how well they felt informed about general CL care and CL-associated ocular complications using a 5-point single-choice Likert scale (good, sufficient, neutral, insufficient, poor). Regarding general CL care, a majority of participants across all four CL types felt either well or sufficiently informed, with 32 of 38 (84.2%) of daily disposable, 44 of 48 (91.7%) reusable SCL, 39 of 40 (97.5%) RCL, and 28 of 30 (93.3%) ScCL wearers. In each of the daily disposable SCL (2.6%), RCL (2.5%), and ScCL (3.3%) subgroups, one participant reported feeling insufficiently or poorly informed. Regarding the participants’ perceived knowledge about CL-associated ocular complications, 18 of 38 (47.4%) of daily disposable and 27 of 48 (56.3%) of reusable SCL wearers felt well or sufficiently informed, whereas this was the case for 32 of 40 (80.0%) of RCL and 24 of 30 (80.0%) ScCL users. Participants who reported feeling insufficiently or poorly informed about ocular complications included 9 of 38 (23.7%) daily disposable SCL, 10 of 48 (20.8%) reusable SCL, 2 of 40 (5.0%) RCL, and 3 of 30 (10.0%) ScCL wearers. Fig. 2 illustrates the participants’ answers in detail.

### Hand hygiene

**Fig. 3:**
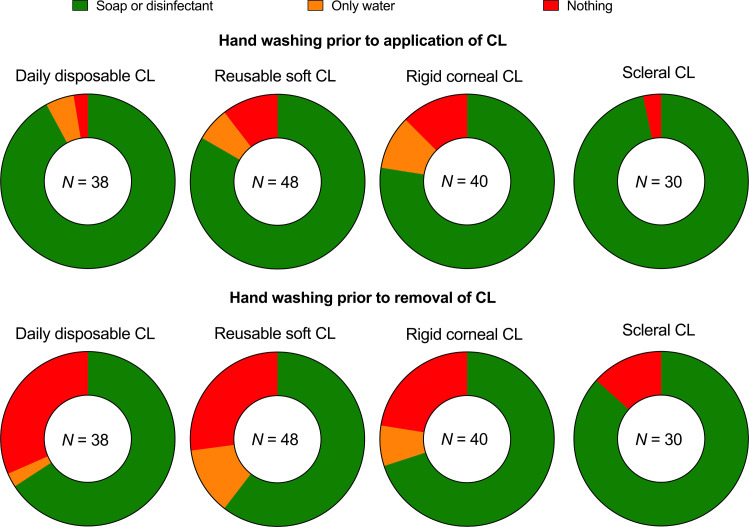
Contact lens subgroup responses regarding handwashing practices before contact lens application (top) and contact lens removal (bottom). CL = contact lens.

Participants were asked whether they wash their hands before applying or removing their CLs. Before inserting their CL, 35 of 38 (92.1%) daily disposable, 40 of 48 (83.3%) reusable SCL, 31 of 40 (77.5%) RCL, and 29 of 30 (96.7%) ScCL wearers reported washing their hands with soap or disinfectant. The remaining participants either skipped handwashing or used only tap water. In the case of CL removal, 25 of 38 (65.8%) daily disposable SCL, 29 of 48 (60.4%) reusable SCL, 28 of 40 (70.0%) RCL, and 26 of 30 (86.7%) ScCL wearers reported cleaning their hands with soap or disinfectant while others only used tap water or did not wash their hands at all. We illustrate the comprehensive results in Fig. 3.

### Wearing behavior

**Fig. 4:**
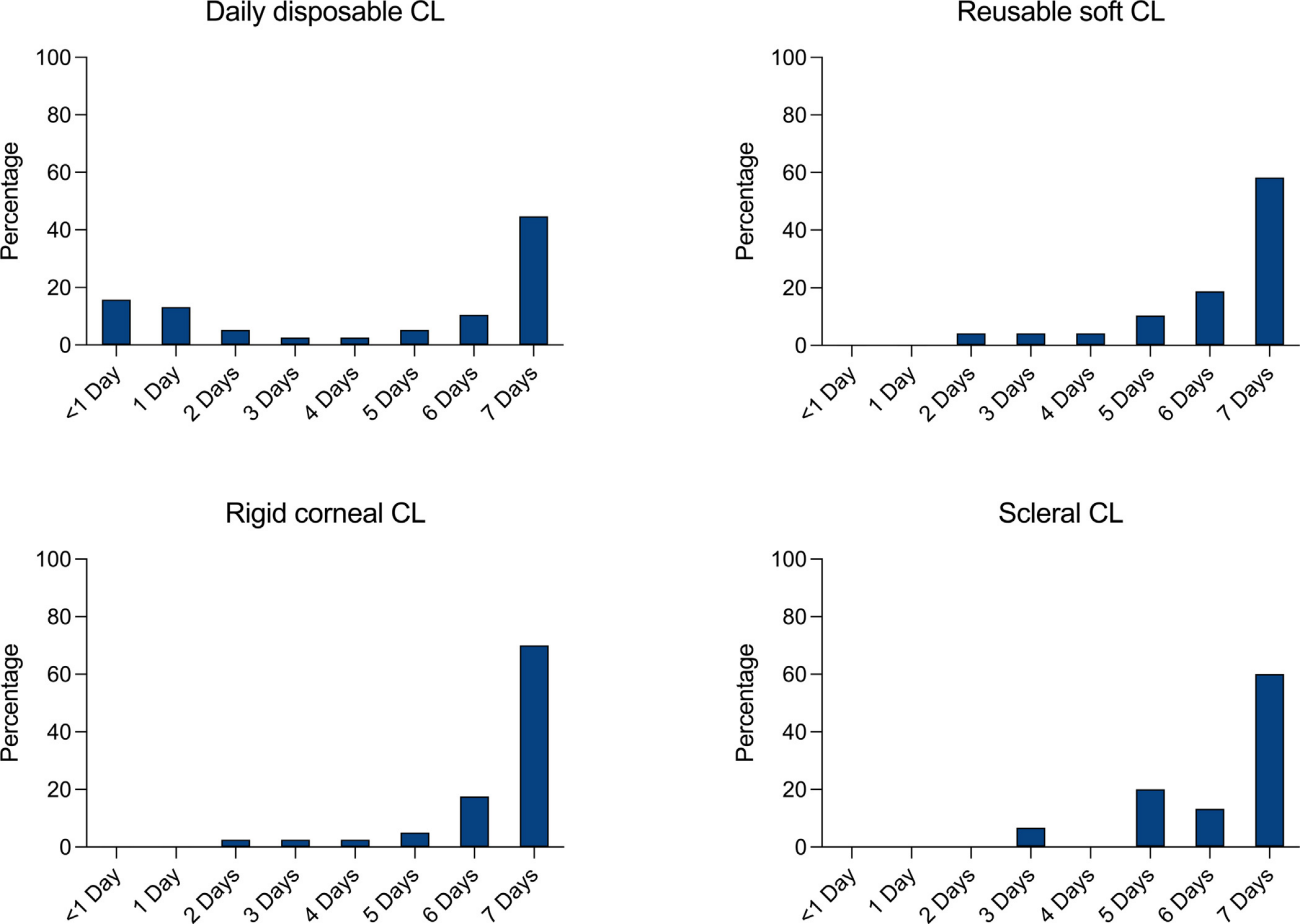
Bar charts displaying the percentage of subgroup participants reporting the number of days per week they wear their contact lenses. CL = contact lens.

**Fig. 5:**
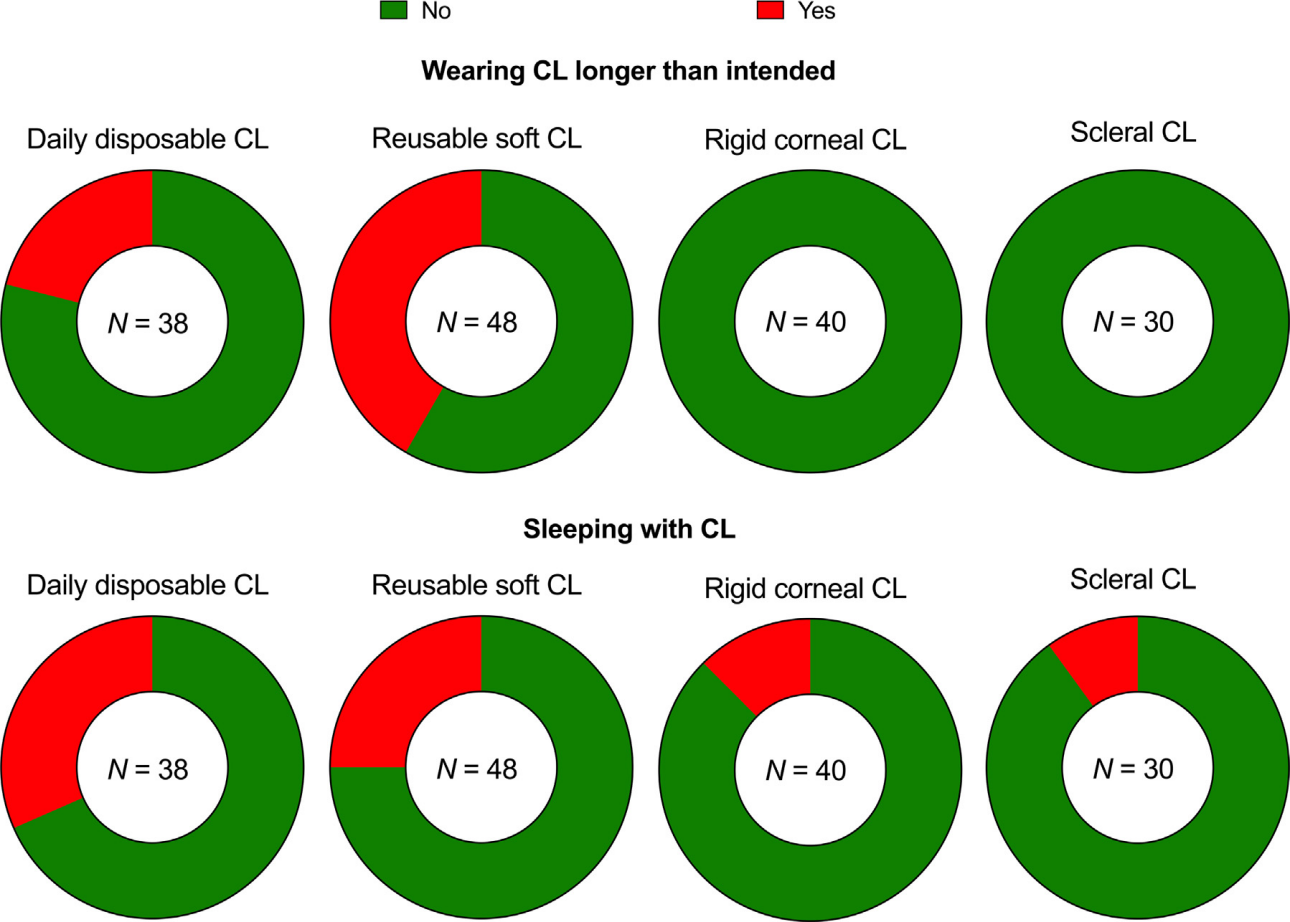
Contact lens wearing behavior. The top donut charts present the responses to whether the participants wear the contact lenses beyond the recommended replacement schedules, and the bottom charts display the answers whether they sleep with the contact lenses. CL = contact lens.

We assessed four distinct aspects of CL wearing behavior: the time spent wearing CLs per day in hours, the number of days worn per week, CL use beyond recommended replacement intervals, and whether participants had ever slept while wearing their CLs. Regarding the number of hours per day, participants wearing daily disposable SCLs reported a median (IQR [range]) wearing time of 12 (8 - 15.25 [1 - 18]) hours per day. Reusable SCL wearers applied their CLs for a median (IQR [range]) time of 14 (12 - 15 [4 - 24]) hours, RCL users for 13.5 (11.25 - 15 [6 - 18]) hours, and the ScCL subgroup for 13.5 (9 - 15 [6 - 22]) hours. Regarding the frequency of CL application per week, daily use was reported by 17 of 38 (44.7%) daily disposable SCL, 28 of 48 (58.3%) reusable SCL, 28 of 40 (70.0%) RCL, and 18 of 30 (60.0%) ScCL wearers. Use of 2–6 days per week was reported by 10 of 38 (26.3%) daily disposable SCL, 20 of 48 (41.7%) reusable SCL, 12 of 40 (30.0%) RCL, and 12 of 30 (40.0%) ScCL users. Only daily disposable SCL wearers reported using CLs ≤1 day per week (11 of 38, 28.9%). Using CLs beyond the recommended replacement schedule was reported by 8 of 38 (21.1%) daily disposable SCL users, with a median (IQR) of 1 (1 - 2) days, and by 20 of 48 (41.7%) reusable SCL wearers with 7 (3.25 - 13) days. No overwear was reported in the RCL or ScCL groups. Sleeping with CLs was reported by 12 of 38 (31.6%) daily disposable SCL, 12 of 48 (25.0%) reusable SCL, 5 of 40 (12.5%) RCL, and 3 of 30 (10.0%) ScCL wearers. Fig. 4, Fig. 5 present further details regarding the CL wearing behavior.

### Exposure to water or saliva

**Fig. 6:**
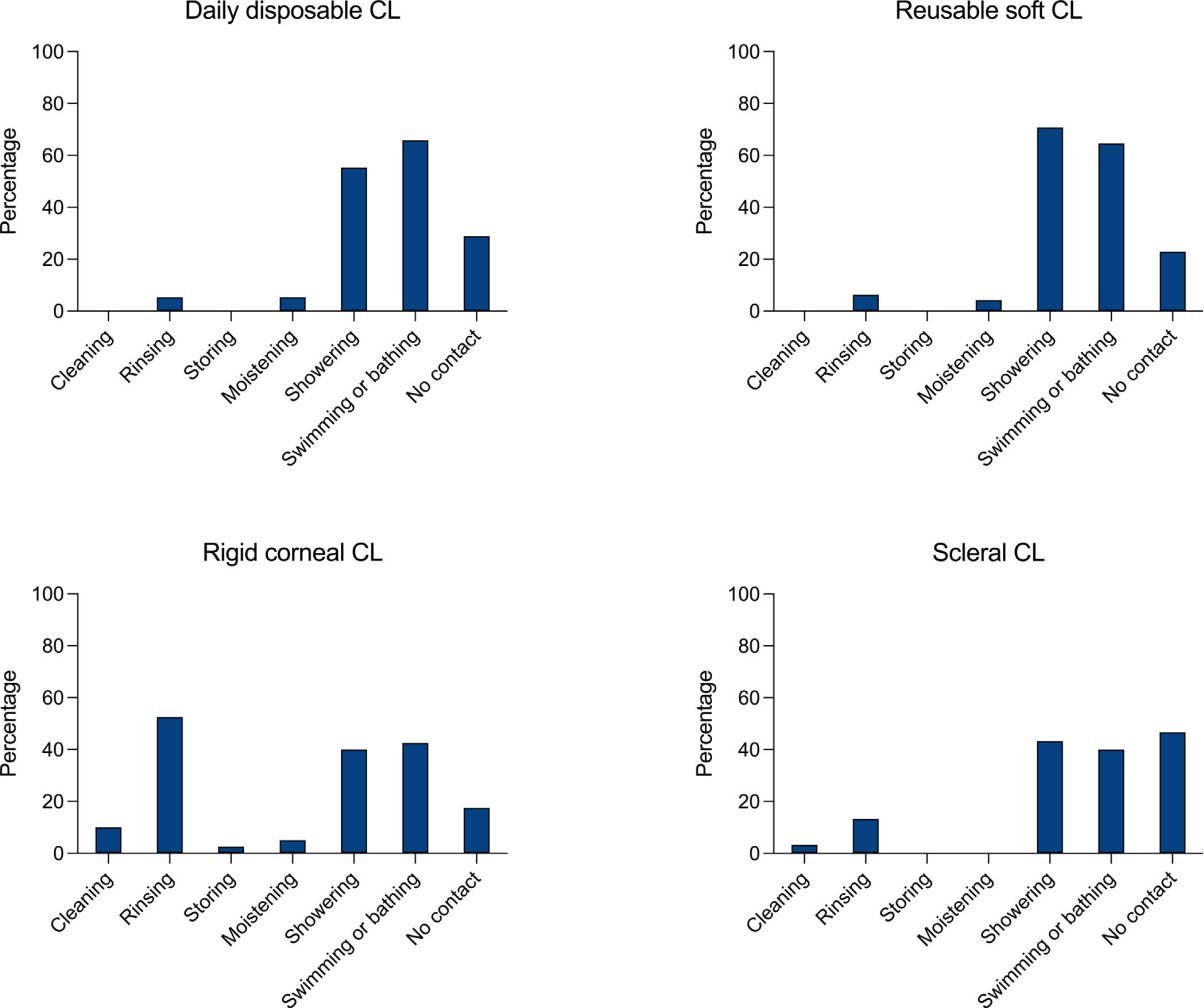
Contact lens exposure to water in different scenarios. The answers were recorded in multiple-choice formats and are displayed according to the contact lens subgroups. CL = contact lens.

**Fig. 7:**
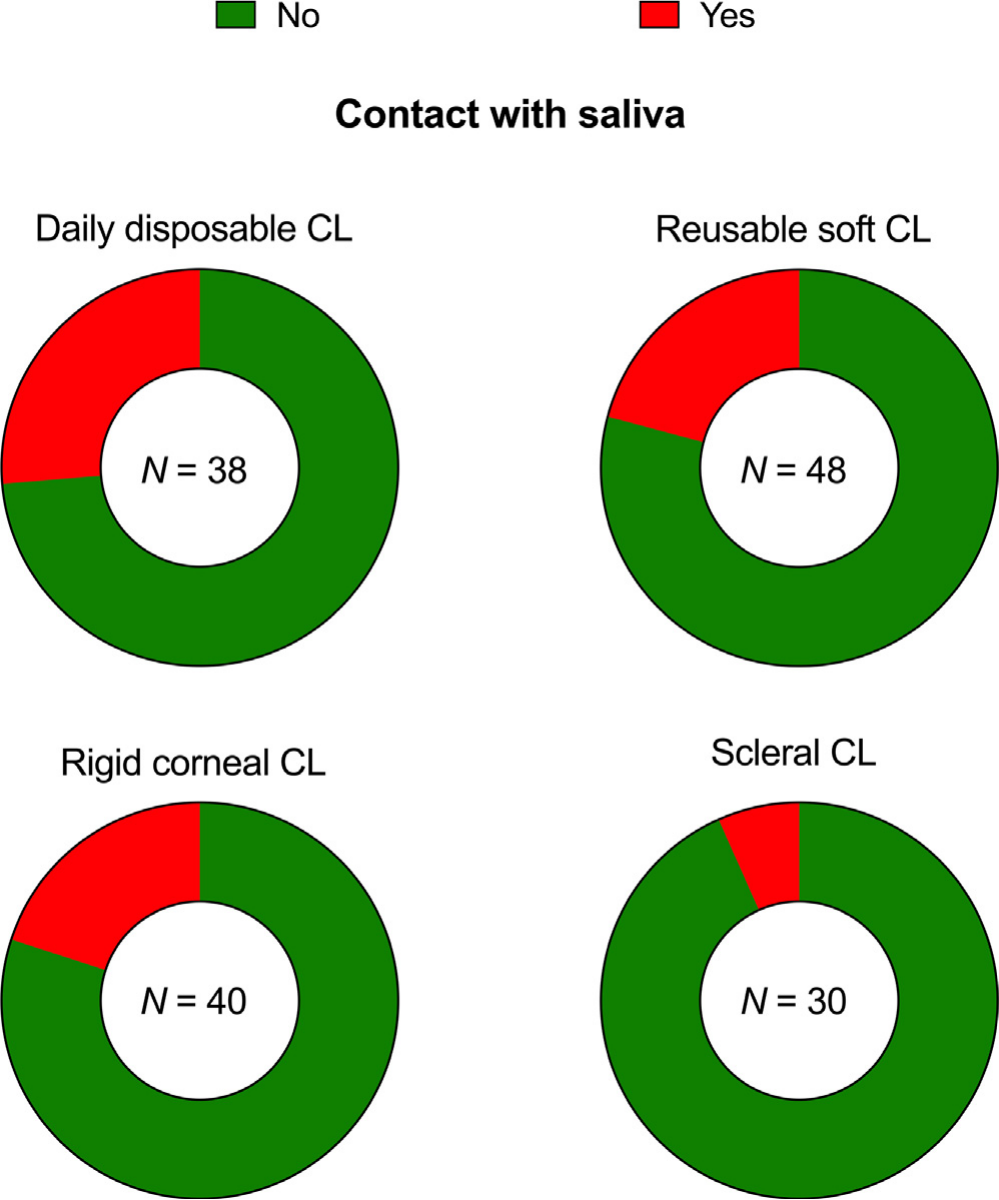
Illustration regarding contact lens exposure to saliva. CL = contact lens.

Participants were asked in a multiple-choice format whether their CLs encountered water in various situations, including rinsing CLs with tap water, showering or swimming while wearing CLs, and moisturizing or cleaning their CLs with saliva. Swimming or bathing with CLs was reported by 25 of 38 (65.8%) daily disposable SCL, 31 of 48 (64.6%) reusable SCL, 17 of 40 (42.5%) RCL, and 12 of 30 (40.0%) ScCL wearers. Showering while wearing CLs was reported by 21 of 38 (55.3%) daily disposable SCL, 34 of 48 (70.8%) reusable SCL, 16 of 40 (40.0%) RCL, and 13 of 30 (43.3%) ScCL users. Rinsing their CLs with tap water was noted by 21 of 40 (52.5%) RCL and 4 of 30 (13.3%) ScCL wearers. Cleaning CLs with tap water was reported by 4 of 40 (10.0%) RCL and 1 of 30 (3.3%) ScCL users. No water contact was reported in any of the questioned scenarios by 11 of 38 (28.9%) daily disposable SCL, 11 of 48 (22.9%) reusable SCL, 7 of 40 (17.5%) RCL, and 14 of 30 (46.7%) ScCL wearers. Moisturizing or cleaning CLs with saliva was reported by 10 of 38 (26.3%) daily disposable SCL, 10 of 48 (20.8%) reusable SCL, 8 of 40 (20.0%) RCL, and 2 of 30 (6.7%) ScCL users. Fig. 6, Fig. 7 show a detailed breakdown of water and saliva exposure.

## Discussion

This observational study provides a comprehensive overview regarding demographics, self-perceived knowledge, hygiene practices, wearing behavior, and exposure to water or saliva of CL wearers in Switzerland across four major CL subtypes. Our findings highlight considerable behavioral differences depending on the lens type and indicate persistent gaps in hygiene and risk awareness, particularly among SCL users. This differentiation is crucial for developing CL-specific educational strategies, as risk profiles may vary depending on the type of CL.^20^^,^^30^^,^^31^

The demographic structure of our cohort reflects a broad user base, with a median age of approximately 40 years and a range between 10 and 82 years. Regarding the investigated types of CLs, the distribution was relatively balanced, with a minor predominance of SCL users. However, with a cumulative proportion of 44.8%, the RCL and ScCL appear overrepresented in this study compared to other prevalence studies, which report a proportion of approximately 10–20%.^32^^,^^33^ This mismatch may be attributable to selection bias, as many of our participants were recruited from a university hospital setting and required RCL or ScCL due to corneal pathologies such as keratoplasty, advanced keratoconus or other severe ocular surface diseases.

The participants’ self-assessed knowledge revealed a contrast between the general CL care and the awareness of CL-related complications. While most participants across all subgroups felt well or sufficiently informed about the CL care, their perceived knowledge of ocular complications was notably lower. Only 47.4% of daily disposable SCL and 56.3% of reusable SCL users considered themselves well or sufficiently informed about possible health implications. In contrast, 80.0% of RCL and ScCL users reported adequate understanding. This gap may reflect systematic differences in clinical supervision by the different CL types. Rigid CLs require professional fitting and regular follow-up by an eye care practitioner, which may improve user education.^25^ In contrast, SCLs are often purchased online or through non-specialized retailers, a practice that has been associated with noncompliant behavior such as infrequent handwashing or exposure to water or saliva.^34^^,^^35^ Beyond education, SCL wearers also require professional oversight to prevent improper fitting, which may lead to an increased risk of infection.^36^ However, direct comparative data on self-reported knowledge and hygiene practices between SCL and rigid CL wearers remain limited. Further research may enable lens-specific educational strategies to enhance patient compliance.

Proper hand hygiene remains a cornerstone of CL safety, as poor handwashing increases the risk of microbial contamination and subsequent keratitis.^37^^,^^38^ While most participants in our study reported washing their hands with soap or disinfectant before inserting their CLs, a considerable proportion did not, particularly among reusable SCL and RCL users. Furthermore, relative to CLs application, the prevalence of hand hygiene before CLs removal declined across all subgroups, with the lowest rates observed among SCL wearers. Improper hand hygiene is a consistently reported significant risk factor for infectious keratitis,^19^^,^^25^^,^^39^ with data indicating a 13-fold risk increase.^40^ Unhygienic handling of CLs also affects the contamination rate of storage cases, especially relevant for reusable CLs.^41^ These findings underscore the need for patient education, reinforcing the importance of hand hygiene during CL care, particularly for reusable CLs.

As CLs act as foreign bodies on the ocular surface, they require proper wearing habits to reduce the risk of complications.^20^^,^^42^^,^^43^ In our cohort, median daily CL wear time and frequency of use were generally high across all CL types. Although data on keratitis risk directly linked to the daily wear time is limited, the literature suggests that wearing comfort declines significantly after 16 h.^44^ In contrast, frequent CL wear of more than four days per week increases the risk for infectious keratitis.^40^ Particularly among SCL users, we observed a high prevalence of overnight lens use and extended use beyond the recommended replacement schedules, both of which are risk factors for infectious keratitis, regardless of the CL material.^20^^,^^24^^,^^31^^,^^45^^,^^46^ Nevertheless, substantial technological advances have been achieved with modern, thin silicone hydrogel CLs with high oxygen permeability, which allow continuous wear for up to 30 days and are marketed as extended wear SCLs.^47^^,^^48^ Despite these developments, extended wear SCLs currently account for only about 10% of the global SCL market.^11^ This may reflect the persistent clinical tendency to discourage overnight wear because of the increased risk of microbial keratitis.^49^ However, extended wear modalities may still offer advantages for selected SCL users, such as those with high refractive errors or demanding work schedules including night shifts. With appropriate patient selection, regular practitioner follow-up, and thorough patient education, extended wear SCL may therefore represent a viable option despite the remaining risk of infectious keratitis.^50^

Further, regarding exposure to potentially contaminated water, 55.3–70.8% of SCL and 40–43.3% of rigid CL users disclosed swimming or showering while wearing their CLs. Contact between the CL and non-sterile water should be avoided, as it represents a significant risk factor for Acanthamoeba keratitis.^32^ This rare but severe ocular infection may lead to a poor visual prognosis.^32^ Additionally, a majority of RCL users reported rinsing their lenses with tap water instead of sterile saline solution, a highly discouraged practice.^51^ The high prevalence of rinsing the RCLs may be due to misinformation or inadequate patient education by eye care providers. Moreover, saliva exposure was also reported among all subgroups, although this cleaning or moisturizing behavior is a less established but theoretically critical practice.^23^ Altogether, future patient information should highlight risk behavior associated with the correct CL cleaning and offer safe options to moisturize the CLs.

Similar to other studies, we also found noncompliant CL behavior in our cohort.^24^^,^^52^^,^^53^ Literature reports that up to 80% of CL-related complications are attributed to user noncompliance^54^; therefore, targeted patient education remains essential for improving ocular safety. Moreover, the complexity of the lens care systems themselves may contribute to complications. There are up to 49 separate steps described for a fully compliant care of reusable CLs.^55^ Even if daily disposable SCLs reduce this burden in theory, our findings indicate that poor hygiene practices also persist among this cohort. Overall, this highlights not only the necessity for further education even in simplified regimens, but also the need for collaboration between industry, patients, and eye care professionals to develop user-friendly technologies that promote safer CL care.^45^

Non-compliance with recommended CL care remains a widespread problem and represents an important modifiable risk factor for CL-related microbial keratitis. Several studies have demonstrated that many CL wearers overestimate their adherence to hygiene recommendations while simultaneously engaging in unsafe behaviors such as improper lens replacements, water exposure or overnight wear.^52^^,^^53^ Improving patient compliance therefore represents a key preventive strategy. Recent literature has shown that simple behavioral interventions, such as visual reminders or targeted educational tools, can significantly reduce risk behaviors such as water exposure and CL case contamination.^56^ In clinical practice, a structured approach combining verbal informing, written instructions such as brochures, and practical demonstrations of proper CL care may improve adherence. Regular follow-up visits with eye care professionals are also essential to reinforce safe practices and to identify non-compliant behaviors early. Particular attention should be paid to SCL users, as overnight wear remains a major risk factor for microbial keratitis.^18^ Clear communication that sleeping while wearing CLs substantially increases infectious risk, together with careful patient selection and close monitoring when extended wear CLs are prescribed, may help reduce the risk of severe complications.^57^

This study has several limitations. First, all qualitative research inherently limits causal inferences at the population level, with each observation given equal consideration, irrespective of its frequency or likelihood of occurrence. Second, recruitment primarily from university hospitals and specialized clinics may have introduced selection bias, with overrepresentation of individuals with complex ocular conditions, such as participants suffering from keratoconus wearing rigid CLs, therefore limiting generalizability. Although limited by our sample size, further analyses of other CL types such as orthokeratology lenses, as well as subgroups separating ametropic and pathological indications would be of great interest for future studies. Third, the reliance on self-reported data introduces the possibility of recall bias and social desirability bias, which may have led participants to underreport noncompliant behaviors or overstate adherence to recommended practices. Fourth, the study did not comprehensively assess all relevant risk behaviors, such as topping off solutions, frequency of CL case replacement, or use of expired disinfecting solutions. Additionally, an independent analysis based on different CL materials was not possible. Further, the risk factors were not weighted according to their clinical relevance. Future research incorporating weighted risk models may enhance the clinical interpretability of these findings. Finally, because the study did not cover patients suffering from infectious keratitis, we could not correlate reported risk behaviors with incidence rates or clinical outcomes. Future research, employing prospective designs and broader recruitment settings, would help validate and expand upon these findings.

In conclusion, we identified substantial variations in hygiene practices, wearing behavior, water and saliva exposure, and risk perception among CL wearers in Switzerland. While rigid CL users demonstrated greater adherence to safety recommendations, SCL wearers, particularly those using daily disposable SCL, engaged in multiple high-risk behaviors. Future interventions should target these subgroups with personalized education, emphasizing the importance of hand hygiene at both insertion and removal, adherence to replacement schedules, and the avoidance of water and saliva exposure. Collaboration between eye care professionals, industry, and regulatory bodies is essential to streamline CL care instructions and eliminate outdated or unsafe recommendations. Such strategies are crucial for mitigating preventable complications and promoting safe CL wear across all user groups. Future studies with larger sample sizes should consider stratified analyses by indication, especially regarding ametropic and pathological cohorts, and include other subgroups such as patients suffering from infectious keratitis or different contact lens modalities such as orthokeratology or extended wear contact CLs.

## Data availability statement

Data will be made available upon request to the corresponding author.

## Informed consent statement

Verbal informed consent was obtained from all participants prior to inclusion. According to local ethical regulations for anonymized survey-based studies, written consent was not required.

## Funding

This study received no external funding.

## Declaration of competing interest

The authors declare no conflicts of interest related to this topic.

## References

[1] Heitz R.F. (1984). The invention of contact lenses by August Müller (1887). Clao J.

[2] McMahon T.T., Zadnik K. (2000). Twenty-five years of contact lenses: the impact on the cornea and ophthalmic practice. Cornea.

[3] Haworth K., Travis D., Leslie L., Fuller D., Pucker A.D. (2023). Silicone hydrogel versus hydrogel soft contact lenses for differences in patient-reported eye comfort and safety. Cochrane Database Syst Rev.

[4] Moreddu R., Vigolo D., Yetisen A.K. (2019). Contact lens technology: from fundamentals to applications. Adv Healthc Mater.

[5] Sharma N., Sah R., Priyadarshini K., Titiyal J.S. (2023). Contact lenses for the treatment of ocular surface diseases. Indian J Ophthalmol.

[6] Morgan P.B., Efron N. (2022). Global contact lens prescribing 2000-2020. Clin Exp Optom.

[7] Efron N., Morgan P.B., Woods C.A., Jones D., Jones L., Nichols J.J. (2024). International trends in prescribing silicone hydrogel contact lenses for daily wear (2000-2023): an update. Cont Lens Anterior Eye.

[8] Dumbleton K., Caffery B., Dogru M., Hickson-Curran S., Kern J., Kojima T. (2013). The TFOS international workshop on contact lens discomfort: report of the subcommittee on epidemiology. Invest Ophthalmol Vis Sci.

[9] Ezinne N.E., Bhattarai D., Ekemiri K.K., Harbajan G.N., Crooks A.C., Mashige K.P. (2022). Demographic profiles of contact lens wearers and their association with lens wear characteristics in Trinidad and Tobago: a retrospective study. PLoS One.

[10] OPTIKSCHWEIZ - Der Verband für Optometrie und Optik: Sehen Schweiz October 2021 [cited 2025 April]. Available from: https://www.optikschweiz.ch/wp-content/uploads/2021/10/OS-Medienmitteilung-210027.pdf.

[11] Morgan B.P .WC., Tranoudis I.G (2022). International contact lens prescribing in 2022. Contact Lens Sprectrum.

[12] Rodriguez-Garcia A. (2021).

[13] Stapleton F., Keay L., Jalbert I., Cole N. (2007). The epidemiology of contact lens related infiltrates. Optom Vis Sci.

[14] Flaxman S.R., Bourne R.R.A., Resnikoff S., Ackland P., Braithwaite T., Cicinelli M.V. (2017). Global causes of blindness and distance vision impairment 1990-2020: a systematic review and meta-analysis. Lancet Glob Health.

[15] Cabrera-Aguas M., Khoo P., Watson S.L. (2022). Infectious keratitis: a review. Clin Exp Ophthalmol.

[16] Ung L., Acharya N.R., Agarwal T., Alfonso E.C., Bagga B., Bispo P.J. (2019). Infectious corneal ulceration: a proposal for neglected tropical disease status. Bull World Health Organ.

[17] Linaburg T.J., Hammersmith K.M. (2024). Contact lens-related corneal infections. Infect Dis Clin North Am.

[18] Maier P., Betancor P.K., Reinhard T. (2022). Contact lens-associated Keratitis-an often underestimated risk. Dtsch Arztebl Int.

[19] Fonn D., Jones L. (2019). Hand hygiene is linked to microbial keratitis and corneal inflammatory events. Cont Lens Anterior Eye.

[20] Stapleton F., Keay L., Edwards K., Naduvilath T., Dart J.K., Brian G. (2008). The incidence of contact lens-related microbial keratitis in Australia. Ophthalmology.

[21] Arshad M., Carnt N., Tan J., Ekkeshis I., Stapleton F. (2019). Water exposure and the risk of contact lens-related disease. Cornea.

[22] Sakr S.I., Nayel A.A., Khattab A.L., Elhamamsy W.M., Abozaid I.A., Awad R. (2024). Impact of contact lens hygiene risk factors on the prevalence of contact lens-related keratitis in Alexandria-Egypt. J Ophthalmic Inflamm Infect.

[23] Ho J.W., Meirick T., SenGupta D.J., Feng S. (2021). Leptotrichia species isolated from a chronic recurrent corneal ulcer. Am J Ophthalmol Case Rep.

[24] Morgan P.B. (2007). Contact lens compliance and reducing the risk of keratitis. Optician.

[25] Fogt J.S., Roth M., Gardner H.P. (2024). How can we better inform patients of the importance of contact lens compliance?: current perspectives. Clin Optom (Auckl).

[26] Çavdarli C., Bayraktar N., Kılıç M. (2021). Survey of hygiene, behaviours, and awareness regarding contact lens wear with conventional and novel questions. Clin Exp Optom.

[27] Dumbleton K.A., Woods C.A., Jones L.W., Fonn D. (2011). The relationship between compliance with lens replacement and contact lens-related problems in silicone hydrogel wearers. Cont Lens Anterior Eye.

[28] Hickson-Curran S., Chalmers R.L., Riley C. (2011). Patient attitudes and behavior regarding hygiene and replacement of soft contact lenses and storage cases. Cont Lens Anterior Eye.

[29] Vasileiou K., Barnett J., Thorpe S., Young T. (2018). Characterising and justifying sample size sufficiency in interview-based studies: systematic analysis of qualitative health research over a 15-year period. BMC Med Res Methodol.

[30] Cheng K.H., Leung S.L., Hoekman H.W., Beekhuis W.H., Mulder P.G., Geerards A.J. (1999). Incidence of contact-lens-associated microbial keratitis and its related morbidity. Lancet.

[31] Dart J.K., Radford C.F., Minassian D., Verma S., Stapleton F. (2008). Risk factors for microbial keratitis with contemporary contact lenses: a case-control study. Ophthalmology.

[32] Ispizua Mendivil E., Durán de la Colina J.A. (2024). Infectious keratitis associated with contact lens wear: REGINFECOR multicenter study. Arch Soc Esp Oftalmol (Engl Ed).

[33] Morgan P.B., Efron N. (2022). Quarter of a century of contact lens prescribing trends in the United Kingdom (1996 - 2020). Cont Lens Anterior Eye.

[34] Supiyaphun C., Jongkhajornpong P. (2021). Contact lens use patterns, behavior and knowledge among university students in Thailand. Clin Ophthalmol.

[35] Naaman N.K., Alharbi S.Y., Khan M.A., Alghamdi S.A. (2022). Compliance with contact lens care and factors driving noncompliance in health-care students in Jeddah, Saudi Arabia. Saudi J Ophthalmol.

[36] Andrew M., Zimmerman A.T., Bailey M.D. (2025). The relationship between soft contact lens adverse events and corneal sagittal depth. Contact Lens and Anterior Eye.

[37] Carnt N., Hoffman J.M., Verma S., Hau S., Radford C.F., Minassian D.C. (2018). Acanthamoeba keratitis: confirmation of the UK outbreak and a prospective case-control study identifying contributing risk factors. Br J Ophthalmol.

[38] Yee A., Walsh K., Schulze M., Jones L. (2021). The impact of patient behaviour and care system compliance on reusable soft contact lens complications. Cont Lens Anterior Eye.

[39] Stapleton F., Naduvilath T., Keay L., Radford C., Dart J., Edwards K. (2017). Risk factors and causative organisms in microbial keratitis in daily disposable contact lens wear. PLoS One.

[40] Lim C.H., Carnt N.A., Farook M., Lam J., Tan D.T., Mehta J.S. (2016). Risk factors for contact lens-related microbial keratitis in Singapore. Eye (Lond).

[41] Wu Y.T., Willcox M., Zhu H., Stapleton F. (2015). Contact lens hygiene compliance and lens case contamination: a review. Cont Lens Anterior Eye.

[42] Fleiszig S.M.J., Kroken A.R., Nieto V., Grosser M.R., Wan S.J., Metruccio M.M.E. (2020). Contact lens-related corneal infection: intrinsic resistance and its compromise. Prog Retin Eye Res.

[43] Lam J.S., Tan G., Tan D.T., Mehta J.S. (2013). Demographics and behaviour of patients with contact lens-related infectious keratitis in singapore. Ann Acad Med Singap.

[44] Call T., Pucker A.D., McGwin G., Franklin Q.X., Logan A. (2023). Real-time ocular comfort reporting in monthly replacement contact lens wearers. Clin Optom (Auckl).

[45] Fleiszig S.M., Evans D.J. (2010). Pathogenesis of contact lens-associated microbial keratitis. Optom Vis Sci.

[46] Schein O.D., Buehler P.O., Stamler J.F., Verdier D.D., Katz J. (1994). The impact of overnight wear on the risk of contact lens-associated ulcerative keratitis. Arch Ophthalmol.

[47] Donshik P.C. (2003). Extended wear contact lenses. Ophthalmol Clin North Am.

[48] Ţălu Ş. (2021). Advanced morphological analysis of siloxane-hydrogel contact lenses. Microsc Res Tech.

[49] Stapleton F., Carnt N. (2012). Contact lens-related microbial keratitis: how have epidemiology and genetics helped us with pathogenesis and prophylaxis. Eye (Lond).

[50] Dart J.K., Stapleton F., Minassian D. (1991). Contact lenses and other risk factors in microbial keratitis. Lancet.

[51] Kilvington S., Gray T., Dart J., Morlet N., Beeching J.R., Frazer D.G. (2004). Acanthamoeba keratitis: the role of domestic tap water contamination in the United Kingdom. Invest Ophthalmol Vis Sci.

[52] Rueff E.M., Wolfe J., Bailey M.D. (2019). A study of contact lens compliance in a non-clinical setting. Cont Lens Anterior Eye.

[53] Bui T.H., Cavanagh H.D., Robertson D.M. (2010). Patient compliance during contact lens wear: perceptions, awareness, and behavior. Eye Contact Lens.

[54] Ky W., Scherick K., Stenson S. (1998). Clinical survey of lens care in contact lens patients. Clao J.

[55] Efron N., Morgan P.B. (2017). Rethinking contact lens aftercare. Clin Exp Optom.

[56] Arshad M., Carnt N., Tan J., Stapleton F. (2021). Compliance behaviour change in contact lens wearers: a randomised controlled trial. Eye (Lond).

[57] Carnt N., Stapleton F. (2016). Strategies for the prevention of contact lens-related acanthamoeba keratitis: a review. Ophthalmic Physiol Opt.

